# Humoral Factors From Musculoskeletal Polytrauma Patients Impair Antibacterial Responses Of Neutrophils *In vitro*

**DOI:** 10.7150/jbji.35424

**Published:** 2019-11-07

**Authors:** Aikaterini Stylianaki, Barbara Stanic, Mario Morgenstern, Geoff R. Richards, Fintan T. Moriarty, Keith Thompson

**Affiliations:** 1 AO Research Institute Davos, Davos, Switzerland.; 2 Department of Orthopaedic and Trauma Surgery, University Hospital Basel, Switzerland.

**Keywords:** polytrauma, neutrophil, antibacterial activity

## Abstract

Polytrauma is associated with increased risk of sepsis, but the risk for implant infection is less clear. Neutrophil antibacterial responses are significantly reduced in polytrauma patients (n= 9, ISS≥15) for at least 5 days compared to healthy controls. Reduced neutrophil activity could influence implant infection in addition to sepsis.

## Introduction

Fracture-related infections (FRI) occur when pathogens breach the protective barrier of the skin and invade the wound, which may occur during trauma, surgery or postoperatively. Incidence of trauma-related FRI varies markedly (1-30%) depending on injury severity 1. Furthermore, FRI is a major economic burden, with recent estimates of $61'000 - $150'972 per infected case 1 and predicted costs of treating FRI expected to exceed $1.6 billion in the United States alone by 2020 2. To successfully establish an infection, any contaminating microorganisms must avoid both prophylactic antibiotic therapy and host immune defenses. Three reasons are believed to largely explain why certain patients succumb to a FRI. Firstly, contaminating bacteria can rapidly adhere to the implanted device or exposed tissues within the wound and form an antibiotic and host defense-resistant biofilm 3-5. Secondly, there is a defect in granulocyte function in the vicinity of the implant, due to the presence of the implant, leading to an inability to phagocytose bacteria 6. Thirdly, vascular access is often compromised by the accompanying soft tissue trauma limiting antibiotic penetration 7 and the host immune response.

Numerous studies have shown that severe trauma itself induces temporary disorders of the immune system 8-11 that involves an early activation of immune system and the development of systemic inflammatory response syndrome (SIRS) and a partially overlapping compensatory anti-inflammatory response syndrome (CARS). In the most severe cases, this response can result in an increased risk of multi-organ failure and sepsis.

The question remains if severe trauma can also influence the effector immune cells required to clear contaminating bacteria from a fracture-site requiring internal stabilization. As the early perioperative period is the critical time for seeding of the implant, any malfunction of first-responding immune cells with direct antibacterial capacity (neutrophils) would be a critical deficit in the fight against FRI. In this study, we sought to investigate the potential inhibitory effects of polytrauma patient sera on neutrophil function by assessing antibacterial responses of a neutrophil-like cell line exposed to polytrauma sera *in vitro*, and by quantifying the levels of serum-resident neutrophil-relevant immunomodulatory cytokines *in vivo* in the early days post-trauma.

## Materials

### Patients

Nine polytraumatized patients admitted to Thriassio General Hospital (Athens, Greece) were included in this study, with full approval of the local hospital ethics committee. Inclusion criteria were: adult polytraumatized patients with an Injury Severity Score (ISS) ≥15; long bone fracture; aged 18-50 years. Exclusion criteria were severely compromised respiratory or circulatory function; sepsis or suspected infection upon admission to hospital. Serum samples (10 ml) were taken upon arrival, and at 1, 3, 5, and 7 days. The median ISS of enrolled patients was 18 (range: 17-50). Control sera were provided by 10 healthy human donors. All serum samples were collected, processed and stored at -80°C at Thriasso General Hospital. Serum was then transferred to ARI Davos on dry ice for subsequent experimentation.

### Cell culture

The human acute myeloid leukemia cell line PLB-985 (from the German Collection of Microorganisms and Cell Cultures (DMZB)) was grown in RPMI-1640 medium containing 10% FBS, 100 U/ml penicillin and 100 μg/ml streptomycin, at 37°C under humidified conditions and 5% CO_2_. Cells were used up to passage 10 before discarding. To induce differentiation into neutrophil-like cells 12, growth medium was supplemented with 1.25% (v/v) DMSO for 5 days before each experiment to generate differentiated PLB-985 cells (dPLB-985), as previously described 13.

### Assessment of phagocytic cell function

The capacity of polytrauma sera for influencing phagocytosis was investigated using pHrodo^TM^ Green *Staphylococcus aureus* BioParticles^TM^ (ThermoFisher, Zürich, Switzerland) and dPLB-985 cells. Cells were incubated for 4h with either polytrauma or control sera prior to incubation with BioParticles for a further 2h period, according to the manufacturer's instructions. Intracellular fluorescence was then assessed using flow cytometric analysis with a FACS Aria III flow cytometer. Data was analysed using FlowJo software (FlowJo LLC, Oregon).

Oxidative burst capacity in dPLB-985 cells was determined using CellROX reagent (ThermoFisher). Following a 4h incubation with polytrauma or control sera, dPLB-985 cells were activated with 100 nM N-formylmethionine-leucyl-phenylalanine (fMLP; Sigma-Aldrich, Buchs, Switzerland) for 5 min to induce reactive oxygen species (ROS) production, prior to incubation with CellROX reagent for a further 30 min. Cellular fluorescence, corresponding to ROS production, was then determined by flow cytometry.

### Cytokine/chemokine determination

Polytrauma and healthy donor sera were assessed for a range of cytokines and chemokines using Luminex Magnetic Bead Panel kits (Merck Millipore, Zug, Switzerland) and a Bio-Plex multiplex system (Bio-Rad), or single/multiplex assay kits (Meso Scale Diagnostics, Rockville) and a MESO QuickPlex SQ 120 imager.

### Statistics

Statistical analysis was performed using GraphPad Prism software. One-way ANOVA (Kruskall-Wallis with Dunn's multiple comparisons test) was performed to compare polytrauma with healthy control values. Results are expressed as mean ± S.D. (phagocytosis and ROS generation) or mean ± range (cytokine quantification). P values of 0.05 or less were considered significant.

## Results

**Figure 1:**
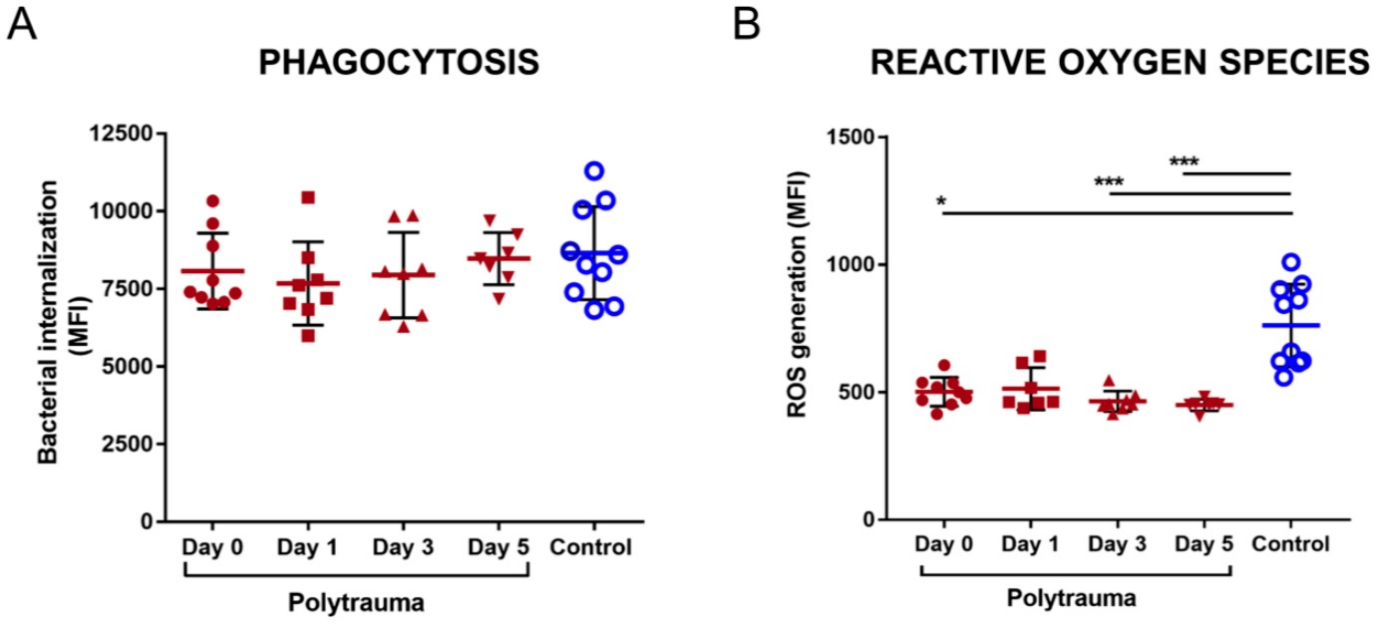
** Effects of polytrauma patient sera on bacterial phagocytosis and generation of reactive oxygen species (ROS) by neutrophil-like cells.** Differentiated PLB-985 cells (dPLB) were pre-treated with cell culture medium containing post-trauma sera or control sera (w/v=10%) for 4h prior to exposure to further stimulation, as further described: **A)** Phagocytosis was triggered by incubation with pHrodo Green-labelled *S. aureus* BioParticles for 2h. Internalization and acidification of the fluorophore in phagosomes results in the generation of a fluorescent signal; **B)** Oxidative burst was measured in dPLB cells by assessing generation of superoxide anions induced by stimulation with 100nM fMLP for 5 min, together with CellROX reagent for a further 30 min incubation. Both assays were quantified using flow cytometric analysis. (Kruskal-Wallis test, Dunn's Multiple Comparison Test, *p<0.05, ***p<0.001).

Of the nine polytrauma patients included in the study, the majorities (8/9) were the result of motor vehicle accidents and the predominant long bone fracture was of the femoral diaphysis (5/9). To investigate the impact of polytrauma sera on phagocytic cell responses, a surrogate neutrophil-like cell line was used, dPLB-985 cells, to minimize the inherent variability in using freshly isolated primary neutrophils from peripheral blood. Following treatment with either healthy donor sera, or with polytrauma sera from any time-points studied (d0-5), there was no difference in the subsequent internalization of pHrodo Green-labelled *S. aureus* by the dPLB-985 cells (Fig. 1A).

Despite having no effect on phagocytic capacity, polytrauma sera markedly decreased fMLP-induced oxidative burst capacity in dPLB-985 cells at all time points tested, reaching significance at d0 (p=0.047), d3 (p<0.001) and d5 (p<0.001) (Fig. 1B).

This reduction in neutrophil oxidative burst capacity was associated with profound decreases in a range of neutrophil-related markers in polytrauma sera, such as IL-8, myeloperoxidase (MPO), MMP-8 and Elastase-2 (Table 1), compared to levels in healthy donors. There was also a significant decrease in circulating levels of TNFα and a trend for increased levels of the immunoregulatory cytokine IL-10 (p=0.058) immediately following trauma (d0), suggestive of a transient systemic immunosuppressed environment resulting from polytraumatic injury. Consistent with this, serum levels of the pro-inflammatory chemokine MIP-1α and HSP-70 levels were significantly decreased, together with an overall trend for decreased serum IL-1α throughout the study period. Increased levels of serum IL-6 were also detected during the first 3 days following trauma but there was no change in the levels of the pro-inflammatory cytokines IL-1β, IL-12p70, CRP, or the chemokines MIP-1β and IP-10 in polytrauma patients compared to healthy donors.

## Discussion

Several clinical research groups have investigated the importance of humoral and cellular factors in the cascade of events subsequent to trauma, including the development of sepsis, multiple organ failure and heterotopic ossification 14. However, there is at present a lack of of data describing the effects of systemic trauma on the risk of developing FRI after internal fracture fixation.

In this study we demonstrate that musculoskeletal polytrauma induces systemic changes in a range of mediators capable of influencing the recruitment and function of phagocytic innate immune cells such as neutrophils. These serum-resident mediators induced functional changes in a neutrophil-like cell line that, although not affecting the phagocytic capacity of these cells, did impair the respiratory burst capacity of these cells, thereby likely diminishing their ability to kill internalized bacteria. Intracellular survival of bacteria such as *S. aureus* is a recognized phenomenon in established bone infection and may also be critical in the initiation of infection. Furthermore, we observed that a variety of mediators influencing neutrophil recruitment and activity were also diminished in polytrauma serum, suggesting a potential systemic deficiency in the antibacterial capacity of neutrophils immediately following trauma.

It has been previously reported that serum from trauma patients can reduce the respiratory burst of neutrophils from healthy donors 15 although the identity of the factor(s) responsible was not confirmed, and the impact of polytrauma serum for affecting phagocytic capacity and phagosomal maturation was not determined. Consistent with this, femoral fracture patients have also been demonstrated to display functional deficits in neutrophil behavior concerning phagocytosis and antibacterial efficacy against common FRI-causative pathogens such as *S. aureus*, *S. epidermidis* and *P. aeruginosa* 16, and serum from these patients was also shown to display diminished neutrophil chemotactic properties compared to healthy donors, consistent with our findings.

The specific mechanism concerning the diminished respiratory burst capacity induced by polytrauma serum is also currently unknown but may involve decreased expression of the oxidase enzyme system components p47-phox and p67-phox in neutrophils, which has been previously demonstrated in patients with burn injuries 17. Although it is currently unknown whether a similar mechanism is responsible for the findings in our study, the observation that orthopedic surgeries, such as hip replacement surgery in elderly patients, decreases oxidative burst capacity in neutrophils 11, this is worthy of further investigation since polytrauma patients frequently require fracture fixation surgery.

## Outlook

Further studies aimed at identifying the temporal nature of polytrauma-induced neutrophil dysfunction and recovery would be necessary before the clinical implications of this work can be considered. This would ideally be determined using a serum marker (or profile of markers), although this requires extensive validation in further studies with larger patient cohort sizes. In addition, the impact of polytrauma serum on primary human neutrophils would of interest, as well as of primary human macrophages, which have been associated with sepsis after internalization.

## Conclusion

This study suggests that polytrauma patient sera contain factors impairing host neutrophil antibacterial efficacy through effects on ROS production, with additional possible negative effects on neutrophil recruitment and/or activity. Although our sample size is small and there are issues of patient heterogeneity, such inhibitory effects of neutrophil function may predispose polytrauma patients to an increased risk of FRI analogous to the established impact on sepsis.

## Figures and Tables

**Table 1:** Quantification of serum-resident factors in polytrauma patients compared to healthy donor sera.

Analyte	Analyte concentration (pg/ml)					
	Polytrauma patients					Healthy controls
	Day 0	Day 1	Day 3	Day 5	Day 7	
IL-1α	6.86 (1.18-36.36)	3.54 (1.18-20.09)	2.50 (1.18-11.74)	2.94 (1.18-13.47)	7.65 (1.18-21.05)	14.68 (1.18-57.9)
IL-1β	0.19 (0.17-0.22)	0.52 (0.13-2.85)	0.21 (0.15-0.34)	0.20 (0.17-0.21)	0.23 (0.16-0.42)	0.62 (0.17-3.31)
IL-6	24.5 (4.39-130.9)	19.8* (4.45-51.6)	27.3 (3.88-180.0)	9.09 (2.46-17.5)	19.6 (2.44-67.0)	5.17 (0.64-18.81)
IL-10	8.37* (0.36-44.6)	2.15 (0.32-8.61)	1.66 (0.26-9.04)	0.64 (0.31-1.42)	1.56 (0.32-5.29)	0.75 (0.23-3.05)
IL-12p70	0.26 (0.07-0.69)	0.19 (0.01-0.48)	0.28 (0.04-0.87)	0.12 (0.02-0.21)	0.23 (0.03-0.45)	0.10 (0.01-0.17)
TNFα	1.48* (1.07-2.12)	2.36 (1.08-5.71)	2.61 (1.76-4.77)	2.56 (1.73-4.20)	2.74 (1.59-4.42)	3.02 (0.95-6.74)
IL-8	20.17 (3.99-54.39)	11.81 (3.51-30.58)	14.39 (1.42-51.54)	10.92 (2.65-26.9)	14.0 (7.54-20.65)	535.0 (0.4-2500)
MIP-1α	3.42*** (2.21-5.21)	3.29*** (1.58-7.64)	4.35* (2.85-6.98)	6.41 (2.62-13.09)	4.88 (3.29-7.52)	43.2 (3.73-191.1)
MIP-1β	29.59 (23.58-35.88)	32.13 (17.45-69.44)	28.89 (18.27-44.25)	28.23 (17.31-45.84)	28.24 (22.82-35.35)	55.25 (0.37-295.6)
IP-10	21.43 (6.98-43.89)	17.16 (5.39-25.85)	51.68 (18.79-196.2)	89.13 (9.24-370.1)	60.63 (8.04-153.9)	22.35 (9.44-33.06)
MCP-4	35.37 (7.59-66.85)	21.32** (7.95-36.09)	26.59 (8.29-50.64)	24.82* (7.95-50.66)	37.57 (21.81-55.22)	58.95 (11.13-121.9)
CRP	67.9 (1.22-146.5)	49.81 (0.82-107.8)	82.55 (3.77-119.7)	65.35 (8.93-137.7)	73.97 (3.63-138.3)	53.74 (2.27-152.2)
MMP-8	1333* (15.16-6099)	956.3** (15.16-3756)	721.7** (15.16-1983)	586.2** (15.16-2050)	1028 (427.5-2509)	25029 (503.3-72188)
HSP70	39.76 (5.11-132.3)	15.3** (2.94-26.39)	21.43* (2.56-52.39)	18.06** (3.64-41.33)	17.37** (4.21-48.89)	83.87 (34.43-170.6)
Elastase-2	10.17 (6.5-18.58)	9.92 (6.5-13.49)	9.02* (5.85-12)	8.60* (5.83-12.58)	10.18 (8.05-13.09)	22.49 (8.72-65.02)
MPO	510.7 (3.42-1302)	607.2 (3.99-2285)	246.0 (3.63-459.5)	117.2* (2.0-279.7)	506.5 (247-1119)	3358 (2.58-13975)

Sera from post-trauma patients or healthy donors were analysed for levels of a range of cytokines, chemokines and other neutrophil-related mediators, using multiplexed assays. Data shown are the mean values, with the range indicated within brackets. (Kruskal-Wallis test, Dunn's Multiple Comparison Test; *p<0.05, ***p<0.001 versus healthy control values).
